# Data extraction methods for systematic review (semi)automation: A living review protocol

**DOI:** 10.12688/f1000research.22781.2

**Published:** 2020-06-08

**Authors:** Lena Schmidt, Babatunde K. Olorisade, Luke A. McGuinness, James Thomas, Julian P. T. Higgins

**Affiliations:** 1 Bristol Medical School, University of Bristol, Bristol, BS8 2PS, UK; 2 UCL Social Research Institute, University College London, London, WC1H 0AL, UK

**Keywords:** Data Extraction, Natural Language Processing, Reproducibility, Systematic reviews, Text mining

## Abstract

**Background:** Researchers in evidence-based medicine cannot keep up with the amounts of both old and newly published primary research articles. Support for the early stages of the systematic review process – searching and screening studies for eligibility – is necessary because it is currently impossible to search for relevant research with precision. Better automated data extraction may not only facilitate the stage of review traditionally labelled ‘data extraction’, but also change earlier phases of the review process by making it possible to identify relevant research. Exponential improvements in computational processing speed and data storage are fostering the development of data mining models and algorithms. This, in combination with quicker pathways to publication, led to a large landscape of tools and methods for data mining and extraction.

**Objective:** To review published methods and tools for data extraction to (semi)automate the systematic reviewing process.

**Methods:** We propose to conduct a living review. With this methodology we aim to do constant evidence surveillance, bi-monthly search updates, as well as review updates every 6 months if new evidence permits it. In a cross-sectional analysis we will extract methodological characteristics and assess the quality of reporting in our included papers.

**Conclusions:** We aim to increase transparency in the reporting and assessment of automation technologies to the benefit of data scientists, systematic reviewers and funders of health research. This living review will help to reduce duplicate efforts by data scientists who develop data mining methods. It will also serve to inform systematic reviewers about possibilities to support their data extraction.

## Introduction

### Background

**Figure 1.**
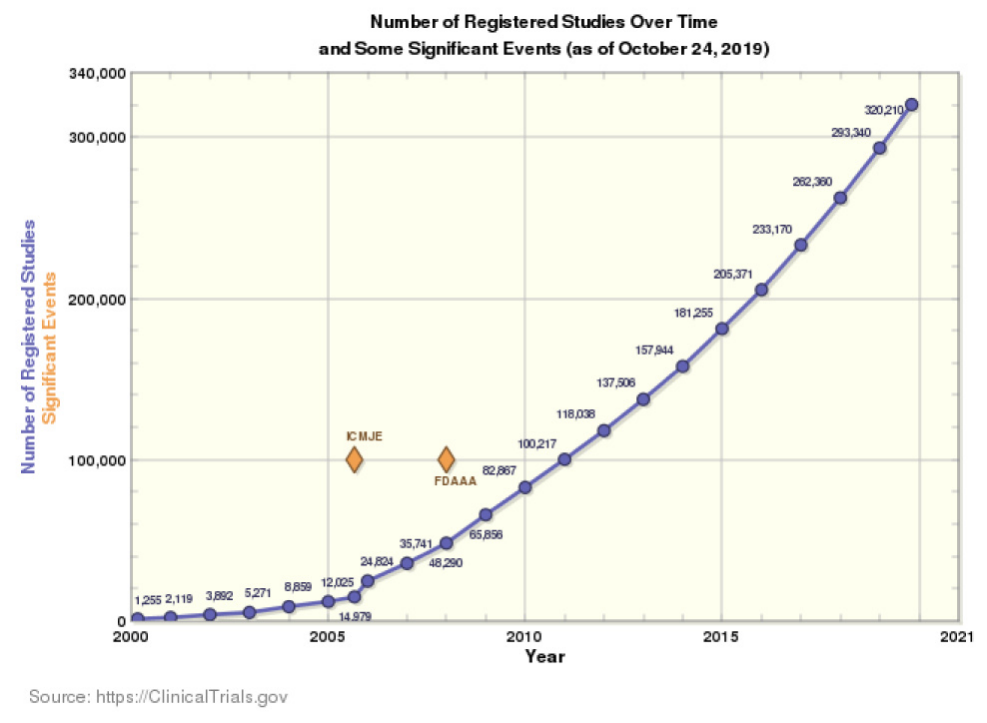
Study registrations on ClinicalTrials.gov show an increasing trend.

Research on systematic review (semi)automation sits at the interface between evidence-based medicine and data science. The capacity of computers for supporting humans increases, along with the development of processing power and storage space. Data extraction for systematic reviewing is a repetitive task. This opens opportunities for support through intelligent software. Tools and methods in this domain frequently focused on automatic processing of information related to the PICO framework (Population, Intervention, Comparator, Outcome). A 2017 analysis of 195 systematic reviews investigated the workload associated with authoring a review. On average, the analysed reviews took 67 weeks to write and publish. Although review size and the number of authors varied between the analysed reviews, the authors concluded that supporting the reviewing process with technological means is important in order to save thousands of personal working hours of trained and specialised staff ^1^. The potential workload for systematic reviewers is increasing, because the evidence base of clinical studies that can be reviewed is growing rapidly ( Figure 1). This entails not only a need to publish new reviews, but also to commit to them and to continually keep the evidence up to date.

### Rapid development in the field of systematic review (semi)automation

Language processing toolkits and machine learning libraries are well documented and available to use free of charge. At the same time, freely available training data make it easy to train classic machine-learning classifiers such as support vector machines, or even complex, deep neural networks such as long short-term memory (LSTM) neural networks. These are reasons why health data science, much like the rest of computer science and natural language processing, is a rapidly developing field. There is a need for fast publication, because trends and state-of-the-art methods are changing at a fast pace. Preprint repositories, such as the arXiv, are offering near rapid publication after a short moderation process rather than full peer review. Consequently, publishing research is becoming easier.

### Why this review is needed

An easily updatable review of available methods and tools is needed to inform systematic reviewers, data scientists or their funders on the status quo of (semi)automated data extraction methodology. For data scientists, it contributes to reducing waste and duplication in research. For reviewers, it contributes to highlighting the current possibilities for data extraction and empowering them to choose the right tools for their task. Currently, data extraction represents one of the most time consuming ^1^ and error-prone (Jones, Remmington, Williamson, Ashby, & Smyth, 2005) elements of the systematic review process, particularly if a large number of primary studies meet the inclusion criteria. Data mining, paralleled by automatic data extraction of relevant data for any specific systematic review project, has the potential to disrupt the traditional systematic reviewing process. This systematic review workflow usually follows the steps of searching, screening, and extracting data. If high-quality and curated data mining results are available then the searching and screening process is likely to change in the future. This review will provide constant surveillance of emerging data extraction tools.

Many systematic reviewers are free to use any tool that is available to them and need sufficient information to make informed decisions about which tools are to be preferred. Our proposed continuous analysis of the available tools will include the final performance metrics that a model achieves, and will also assess dimensions such as transparency of methods, reproducibility, and how these items are reported. Reported pitfalls of applying health data science methods to systematic reviewing tasks will be summarised to highlight risks that current, as well as future, systems are facing. Reviewing the available literature on systematic review automation is one of many small steps towards supporting evidence synthesis of all available medical research data. If the evidence arising from a study is never reviewed, and as a result never noticed by policy makers and providers of care, then it counts towards waste in research.

### Aims of this review

This review aims to:

1. Review published methods and tools aimed at automating or semi-automating the process of data extraction in the context of a systematic review of medical research studies.
2. Review this evidence in the scope of a living review, keeping information up to date and relevant to the challenges faced by systematic reviewers at any time.

Our objectives are three-fold. First we want to examine the methods and tools from the data science perspective, seeking to reduce duplicate efforts, summarise current knowledge, and encourage comparability of published methods. Second, we seek to highlight contributions of methods and tools from the perspective of systematic reviewers who wish to use (semi)automation for data extraction: what is the extent of automation?; is it reliable?; and can we identify important caveats discussed in the literature, as well as factors that facilitate the adoption of tools in practice?

### Related research

We have identified three previous reviews of tools and methods, two documents providing overviews and guidelines relevant to our topic, and an ongoing effort to characterise published tools for different parts of the systematic reviewing process with respect to interoperability and workflow integration. In 2014, Tsafnat *et al.* provided a broad overview on automation technologies for different stages of authoring a systematic review ^2^. O'Mara-Eves *et al.* published a systematic review focusing on text-mining approaches in 2015. It includes a summary of methods for the evaluation of systems (such as recall, F1 and related scores). The reviewers focused on tasks related to PICO classification and supporting the screening process ^3^. In the same year, Jonnalagadda *et al.* described methods for data extraction, focusing on PICOs and related fields ^4^.

These reviews present an overview of classical machine learning and NLP methods applied to tasks such as data mining in the field of evidence-based medicine. At the time of publication of these documents, methods such as topic modelling (Latent Dirichlet Allocation) and support vector machines constituted the state-of-the art for language models. The age of these documents means that the latest static or contextual embedding-based and neural methods are not included. These modern methods, however, are used in contemporary systematic review automation software ^5^.

Marshall and Wallace (2019) ^6^ present a more recent overview of automation technologies, with a focus on availability of tools and adoption into practice. They conclude that tools facilitating screening are widely accessible and usable, while data extraction tools are sill at piloting stages or require higher amounts of human input.

Beller *et al.* ^7^ present a brief overview of tools for systematic review automation. They discuss principles for systematic review automation from a meeting of the International Collaboration for the Automation of Systematic Reviews (ICASR). They highlight that low levels of funding, as well as the complexity of integrating tools for different systematic reviewing tasks have led to many small and isolated pieces of software. A working group formed at the ICASR 2019 Hackathon is compiling an overview of tools published on the Systematic Review Toolbox website ^8^. This ongoing work is focused on assessing maintenance status, accessibility and supported reviewing tasks of 120 tools that can be used in any part of the systematic reviewing process as of November 2019.

## Protocol

### Prospective registration of this review

We registered this protocol via OSF ( https://doi.org/10.17605/OSF.IO/ECB3T). PROSPERO was initially considered as platform for registration, but it is limited to reviews with health related outcomes.

### Choosing to maintain this review as a living review

The challenges highlighted in the previous section create several problems. A large variety of approaches and different means of expressing results creates uncertainty in the existing evidence. At the same time, new evidence is being published constantly. Rapid means of publications necessitate a structured, but at the same time easily updatable review of published methods and tools in the field. We therefore chose a living review approach as the updating strategy for this review.

### Search and updates

**Figure 2.**
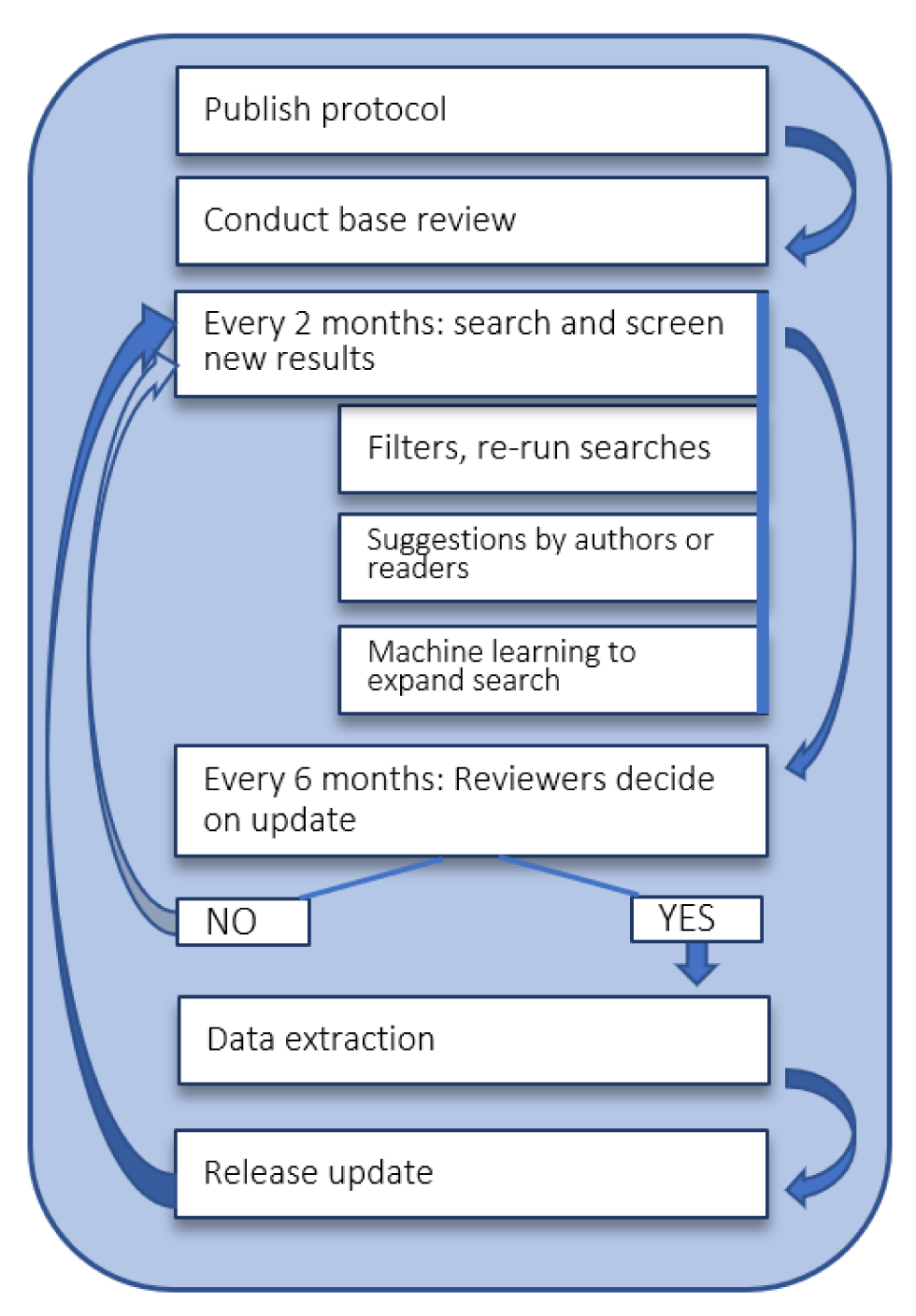
Continuous updating of the living review.

For literature searches and updates we follow the living review recommendations published by Elliott *et al.* ^9^ and Brooker *et al.* ^10^, as well as F1000Research guidelines for projects that are included in their living evidence collection. We plan to run searches for new studies every second month. This will also include screening abstracts of the newly retrieved reports. The bi-monthly interval for screening was chosen because we expect no sudden rise in relevant publications that could justify daily, weekly or monthly screening. The review itself will be updated every six months, providing that a sufficient quantity of new records are identified for inclusion. As a threshold for updating, we plan to use 10 new records, but we will consider updating the review earlier if new impactful evidence is published. We define impactful evidence as, for example, the publication of a tool that is immediately accessible to systematic reviewers and offers substantial automation of the data extraction process, or a tool that aims to change the traditional SR workflow. Figure 2 describes the anticipated reviewing process in more detail.

Our Medline search strategy was developed with the help of an information specialist. Due to the interdisciplinary topic of this review, we plan to search bibliographic databases related to both medicine and computer science. These include Medline via Ovid and Web of Science Core Collection, as well as the computer science arXiv and the DBLP computer science bibliography. We aim to retrieve publications related to two clusters of search terms. The first cluster includes computational aspects such as data mining, while the second cluster identifies publication related to systematic reviews. The Medline search strategy is provided as *Extended data* ^11^. We aim to adapt this search strategy for conducting searches in all mentioned databases. Previous reviews of data mining in systematic reviewing contexts identified the earliest text mining application in 2005 ^3, 4^. We therefore plan to search all databases from this year on. In a preliminary test our search strategy was able to identify 4320 Medline records, including all Medline-indexed records included by O’Mara-Eves *et al.* ^3^. We plan to search the Systematic Review Toolbox website for further information on any published or unpublished tools ^8^.

### Workflow and study design

All titles and abstracts will be screened independently by two reviewers. Any differences in judgement will be discussed, and resolved with the help of a third reviewer if necessary. The process for assessing full texts will be the same. Data extraction will be carried out by single reviewers, and random 10% samples from each reviewer will be checked independently. If needed, we plan to contact the authors of reports for clarification or further information. In the base review, as well as in every published update, we will present a cross-sectional analysis of the evidence from our searches. This analysis will include the characteristics of each reviewed method or tool, as well as a summary of our findings. In addition, we will assess the quality of reporting at publication level. This assessment will focus on transparency, reproducibility and both internal and external validity of the described data extraction algorithms. If we at any point deviate from this protocol, we will discuss this in the final publication.

All search results will be de-duplicated and managed with EndNote. The screening and data extraction process will be managed with the help of Abstrackr ^12^ and customised data extraction forms in Excel. All data, including bi-monthly screening results, will be continuously available on our Open Science Framework (OSF) repository, as discussed in the *Data availability* section.

### Which systematic reviewing tasks are supported by the methods we review

Tsafnat *et al.* ^2^ categorised sub-tasks in the systematic reviewing process that contained published tools and methods for automation. In our overview, we follow this categorisation and focus on tasks related to data retrieval. More specifically, we will focus on software architectures that receive as input a set of full texts or abstracts of clinical trial reports. Report types of interest are randomised controlled trials, cohort, or case-control studies. As output, the tools of interest should produce structured data representing features or findings from the study described. A comprehensive list with data fields of interest can be found in the supplementary material for this protocol.

### Eligibility criteria

**Eligible papers**

- We will include full text publications that describe an original natural language processing approach to extract data related to systematic reviewing tasks. Data fields of interest are adapted from the *Cochrane Handbook for Systematic Reviews of Interventions* ^13^, and defined in the *Extended data* ^11^. We will include the full range of natural language processing (NLP) methods, including for example regular expressions, rule-based systems, machine learning, and deep neural networks.
- Papers must describe a full cycle of implementation and evaluation of a method.
- We will include reports published from 2005 until the present day, similar to O’Mara-Eves *et al.* ^3^ and Jonnalagadda *et al.* ^4^. We will translate non-English reports where feasible.
- The data that that included papers use for mining must be texts from randomised controlled trials, comparative cohort studies or case control studies in the form of abstracts, conference proceedings, full texts or part of the text body.

**Ineligible papers**

We will exclude papers reporting:

- methods and tools related solely to image processing and importing biomedical data from PDF files without any NLP approach, including data extraction from graphs;
- any research that focuses exclusively on protocol preparation, synthesis of already extracted data, write-up, pre-processing of text and dissemination will be excluded;
- methods or tools that provide no natural language processing approach and offer only organisational interfaces, document management, databases or version control; or
- any publications related to electronic health reports or mining genetic data will be excluded.

### Key items of interest

**Primary:**

1. Machine learning approaches used
2. Reported performance metrics used for evaluation
3. Type of data
  - Scope: Abstract, conference proceeding, or full text
  - Target design: Randomised controlled trial, cohort, case-control
  - Type of input: The input data format, for example data imported as structured result of literature search (e.g. RIS), APIs, from PDF or text files.
  - Type of output: In which format are data exported after the extraction, for example as text file.

**Secondary:**

1. Granularity of data mining: Does the system extract specific entities, sentences, or larger parts of text?
2. Other reported metrics, such as impacts on systematic review processes (e.g. time saved during data extraction).

**Assessment of the quality of reporting:** We will extract information related to the quality of reporting and reproducibility of methods in text mining ^14^. The domains of interest, adapted for our reviewing task, are listed in the following.

1. Reproducibility:
  - Are the sources for training/testing data reported?
  - If pre-processing techniques were applied to the data, are they described?
2. Transparency of methods:
  - Is there a description of the algorithms used?
  - Is there a description of the dataset used and of its characteristics?
  - Is there a description of the hardware used?
  - Is the source code available?
3. Testing:
  - Is there a justification/an explanation of the model assessment?
  - Are basic metrics reported (true/false positives and negatives)?
  - Does the assessment include any information about trade-offs between recall and precision (also known as sensitivity and positive predictive value)?
4. Availability of the final model or tool:
  - Can we obtain a runnable version of the software based on the information in the publication?
  - Persistence: is the dataset likely to be available for future use?
  - Is the use of third-party frameworks reported and are they accessible?
5. Internal and external validity of the model:
  - Does the dataset or assessment measure provide a possibility to compare to other tools in same domain?
  - Are explanations for the influence of both visible and hidden variables in the dataset given?
  - Is the process of avoiding over- or underfitting described?
  - Is the process of splitting training from validation data described?
  - Is the model’s adaptability to different formats and/or environments beyond training and testing data described?
6. Other:
  - Does the paper describe caveats for using the method?
  - Are sources of funding described?
  - Are conflicts of interest reported?

### Dissemination of information

We plan to publish the finished review, along with future updates, via F1000Research.

All data will be available via a project on Open Science Framework (OSF): https://osf.io/4sgfz/ (see *Data availability*).

### Study status

Protocol published. We did a preliminary Medline search as described in this protocol and the supplementary material. The final search, including all additional databases, will be conducted as part of the full review.

## Data availability

### Underlying data

No underlying data are associated with this article.

### Extended data

Open Science Framework: Data Extraction Methods for Systematic Review (semi)Automation: A Living Review / Protocol. https://doi.org/10.17605/OSF.IO/ECB3T ^11^

This project contains the following extended data:

- Additional_Fields.docx (overview of data fields of interest for text mining in clinical trials)
- Search.docx (additional information about the searches, including full search strategies)

### Reporting guidelines

Open Science Framework: Data Extraction Methods for Systematic Review (semi)Automation: A Living Review / Protocol. https://doi.org/10.17605/OSF.IO/ECB3T ^11^

Data are available under the terms of the Creative Commons Attribution 4.0 International (CC BY 4.0) data waiver.
