# Supplementary material for: Data extraction methods for systematic review (semi)automation: A living review protocol
Source: F1000Res. 2020 Jun 8;9:210. Originally published 2020 Mar 25. [Version 2] doi: 10.12688/f1000research.22781.2 (PMC7338918; doi:10.12688/f1000research.22781.2)
Supplement: Peer review history

# Peer review: Reviewer response for version 2

**DOI:** 10.5256/f1000research.27003.r64379

**Published:** 2020-07-06

**Authors:** Matt Carter

**Affiliations:** 1 Bond University Centre for Research in Evidence-Based Practice, Bond University, Robina, Australia

No further comments.

Is the study design appropriate for the research question?

Yes

Is the rationale for, and objectives of, the study clearly described?

Yes

Are sufficient details of the methods provided to allow replication by others?

Yes

Are the datasets clearly presented in a useable and accessible format?

No

Reviewer Expertise:

Automation of Systematic Reviews

I confirm that I have read this submission and believe that I have an appropriate level of expertise to confirm that it is of an acceptable scientific standard.

# Peer review: Reviewer response for version 1

**DOI:** 10.5256/f1000research.25153.r62963

**Published:** 2020-05-18

**Authors:** Matt Carter

**Affiliations:** 1 Bond University Centre for Research in Evidence-Based Practice, Bond University, Robina, Australia

I believe that the research proposal clearly lays out its objectives and aims. Although there are some minor edits as per the below list:

1. The abstract should specifically mention PICO data extraction rather than data extraction generally. The "Aims" section outlines this but seems to contradict with the more general "Introduction / Objective" section.
2. While the Tsafnat *et al.* workflow is considered the first, other papers such as Clark *et al.* (2019) or Macleod *et al.* for animal testing are a little more current and also fix some of the gaps with Tsafnat. Since there are a few in this area now a reason should be given for the preference.
3. "We plan to run searches for new studies every second month" - There is currently an effort to get this adjusted to bi-monthly as you suggest but the guidelines specify that this should be monthly. Perhaps just a quick sentence acknowledging this and saying that it is excessive for the subject area?
4. The generated search strategy has a specific filter for English / German papers which seems to contradict the more general phrasing of "We will translate non-English reports where feasible".
5. Data sets are obviously not provided as this is an ongoing paper.

Is the study design appropriate for the research question?

Yes

Is the rationale for, and objectives of, the study clearly described?

Yes

Are sufficient details of the methods provided to allow replication by others?

Yes

Are the datasets clearly presented in a useable and accessible format?

No

Reviewer Expertise:

Automation of Systematic Reviews

I confirm that I have read this submission and believe that I have an appropriate level of expertise to confirm that it is of an acceptable scientific standard, however I have significant reservations, as outlined above.

# Response

**Published:** 2020-05-29

**Authors:** Lena Schmidt

**Affiliations:** University of Bristol, UK

*“I believe that the research proposal clearly lays out its objectives and aims. Although there are some minor edits as per the below list: *

1. *The abstract should specifically mention PICO data extraction rather than data extraction generally. The "Aims" section outlines this but seems to contradict with the more general "Introduction / Objective" section.”*

Thank you for pointing this out, we have made some changes to the abstract and to the aims. Firstly, in the abstract we clarified that the data extraction goal is wider (not limited to PICO, it included fields that are generally of interest in systematic reviews as defined in the Cochrane Handbook). We have made changes in the aims section to reflect that, and to make it more consistent throughout. In the method section, we deleted the section “Objectives” and summarised everything under “Aims of this review” in the introduction.

1. *“While the Tsafnat et al. workflow is considered the first, other papers such as Clark et al. (2019) or Macleod et al. for animal testing are a little more current and also fix some of the gaps with Tsafnat. Since there are a few in this area now a reason should be given for the preference.”*
*  *

In response to this comment we added a more recent paper review paper to our summary of related research (Marshall and Wallace, 2019). The related research was chosen because it was either in the form of a systematic review or it was an overview that is directly related to our topic of interest. We also added an additional explanation for our preference to focus on the data extraction stage of the systematic review process in response to this, and also a previous peer review comment *. *To summarise this quickly, data extraction is one of the most time-consuming and error-prone tasks in the systematic reviewing process. By reviewing automation of data extraction, we aim to summarise the current knowledge. Furthermore, the area of data extraction has future potential to disrupt the “traditional” systematic review process – if data are extracted and well classified centrally then the searching and screening workflow can change as well.

1. *“"We plan to run searches for new studies every second month" - There is currently an effort to get this adjusted to bi-monthly as you suggest but the guidelines specify that this should be monthly. Perhaps just a quick sentence acknowledging this and saying that it is excessive for the subject area?”*
*  *

Thank you, yes. We have added a statement: “The bi-monthly interval for screening was chosen because we expect no sudden rise in relevant publications that could justify daily, weekly or monthly screening”. Furthermore, we added a statement defining impactful research that would lead to a review update even if the threshold of new studies is not met (please see reply to the first peer review for reference)

1. *“The generated search strategy has a specific filter for English / German papers which seems to contradict the more general phrasing of "We will translate non-English reports where feasible".*

Thank you, this item was unclear. This initial draft of the search strategy was created for protocol publication and will be minimally altered for the full search and when the remaining databases are searched. Then it will be impossible to use a language filter on most databases. The initial Medline search specifically included German studies because they are feasible to assess, and for any other database search we have no language restrictions. The process of searching all databases will be described in detail in the full review.

1. *“Data sets are obviously not provided as this is an ongoing paper.”*

Thank you for your peer review and for helping us to improve the quality of our paper.

# Peer review: Reviewer response for version 1

**DOI:** 10.5256/f1000research.25153.r61722

**Published:** 2020-05-01

**Authors:** Emma McFarlane

**Affiliations:** 1 National Institute for Health and Care Excellence, Manchester, UK

This protocol outlines a project to review methods and tools for data extraction to help automate a step in the systematic reviewing process. This will be done in the context of a living systematic review with the aim of providing guidance to reviewers who may want to semi-automate their work.

 The objectives of the study are described clearly and are set within the current context of increasing publications. However, it would be helpful, as part of the aim and purpose of the study, to note why the focus is around the data extraction stage specifically.

 The methods of the study are described in enough detail to be replicated. In terms of the methods, can the authors double check the searching approach for accuracy? The abstract notes searches will be conducted monthly whereas the body of the protocol states every two months. The authors indicated they will update their systematic review when evidence expected to impact is identified however, it would be helpful to include some detail to note how impactful evidence will be defined for people doing similar work. 

 A comment from the authors about their choice of study design for the included papers would be helpful. Identifying RCTs in the context of data science is likely to be challenging so it would be interesting to understand the expectations about the evidence base and how study design could link to the point about impact on the results of the review and need to update.

 The outcomes listed in the protocol appear to be comprehensive. However, it is not clear if consideration was given to accuracy of the tools identified as that could link to the objective of identifying whether automation is reliable. 

 Overall, this appears to be an interesting study straddling the fields of systematic reviewing and data science.

Is the study design appropriate for the research question?

Yes

Is the rationale for, and objectives of, the study clearly described?

Yes

Are sufficient details of the methods provided to allow replication by others?

Yes

Are the datasets clearly presented in a useable and accessible format?

Not applicable

Reviewer Expertise:

I am currently conducting a systematic review of automation in systematic reviewing or guideline development. I am also working on a research project on machine learning within the context of guideline recommendations.

I confirm that I have read this submission and believe that I have an appropriate level of expertise to confirm that it is of an acceptable scientific standard.

# Response

**Published:** 2020-05-29

**Authors:** Lena Schmidt

**Affiliations:** University of Bristol, UK

*Thank you for providing this very helpful peer review. We tried to address the concerns below:*

**

*“This protocol outlines a project to review methods and tools for data extraction to help automate a step in the systematic reviewing process. This will be done in the context of a living systematic review with the aim of providing guidance to reviewers who may want to semi-automate their work.”*

*“The objectives of the study are described clearly and are set within the current context of increasing publications. However, it would be helpful, as part of the aim and purpose of the study, to note why the focus is around the data extraction stage specifically.”*

 Thank you for pointing this out. In the current revision we added more details about why we chose to focus on data extraction. To summarise this quickly, data extraction is one of the most time-consuming and error-prone tasks in the systematic reviewing process. By reviewing automation of data extraction, we aim to summarise the current knowledge. Furthermore, the area of data extraction has future potential to disrupt the “traditional” systematic review process – if data are extracted and well classified centrally then the searching and screening workflow can change as well.

*“The methods of the study are described in enough detail to be replicated. In terms of the methods, can the authors double check the searching approach for accuracy? The abstract notes searches will be conducted monthly whereas the body of the protocol states every two months. “*

 We addressed this, thank you. New articles will be screened every two months.

*“The authors indicated they will update their systematic review when evidence expected to impact is identified however, it would be helpful to include some detail to note how impactful evidence will be defined for people doing similar work.“*

 Thank you, this was not clear previously, and we added some further explanation: “We define impactful evidence as, for example, the publication of a tool that is immediately accessible to systematic reviewers and offers substantial automation of the data extraction process, or a tool that aims to change the traditional SR workflow”

*“A comment from the authors about their choice of study design for the included papers would be helpful. Identifying RCTs in the context of data science is likely to be challenging so it would be interesting to understand the expectations about the evidence base and how study design could link to the point about impact on the results of the review and need to update.”*

*We clarified that we are open to include any paper, as long as the paper reports an automation technology for systematic reviewing that processes texts from clinical studies (not electronic health reports). To clarify further, the texts that the technologies process are likely to be RCT reports, but we are not looking to identify RCTs that compare data extraction tools.*

*“The outcomes listed in the protocol appear to be comprehensive. However, it is not clear if consideration was given to accuracy of the tools identified as that could link to the objective of identifying whether automation is reliable.“*

 Thank you for this comment. In response we clarified that we will extract the reported performance metrics. We do not plan to rank tools based on these metrics because results can vary throughout different datasets. Instead, we aim to assess quality of reporting (reproducibility, transparency…) in detail.

*“Overall, this appears to be an interesting study straddling the fields of systematic reviewing and data science.”*

 Thank you very much for your review, it was very helpful in improving the protocol.
